# Supplementary material for: From sequence to enzyme mechanism using multi-label machine learning
Source: BMC Bioinformatics. 2014 May 19;15:150. doi: 10.1186/1471-2105-15-150 (PMC4229970; doi:10.1186/1471-2105-15-150)
Supplement: Additional file 2 — Java code of ml2db. Additional file ml2db_code.tar.gz contains the Java source code to run the multi-label machine learning experiments and save the results to database. The code’s Javadoc is included. [file 1471-2105-15-150-S2.zip › additional file 2/ml2db/utils/doc/edu/cornell/lassp/houle/RngPack/package-use.html]

Uses of Package edu.cornell.lassp.houle.RngPack


JavaScript is disabled on your browser.


- Overview
- Package
- Class
- Use
- Tree
- Deprecated
- Index
- Help

- Prev
- Next

- Frames
- No Frames

- All Classes

# Uses of Package edu.cornell.lassp.houle.RngPack

- Packages that use edu.cornell.lassp.houle.RngPack

  | Package | Description |
  | --- | --- |
  |  |  |
  | --- | --- |
  | cern.jet.random |  |
  | cern.jet.random.engine |  |
  | edu.cornell.lassp.houle.RngPack |  |
- Classes in edu.cornell.lassp.houle.RngPack used by cern.jet.random

  Class and Description

  RandomElement

  RandomElement is an abstract class that encapsulates uniform random number
  generators.
- Classes in edu.cornell.lassp.houle.RngPack used by cern.jet.random.engine

  Class and Description

  RandomElement

  RandomElement is an abstract class that encapsulates uniform random number
  generators.

  RandomSeedable

  `RandomSeedable` is an abstract class that extends the
  `RandomElement` class to include the ability to automatically
  generate a valid `long` seed from the clock.
- Classes in edu.cornell.lassp.houle.RngPack used by edu.cornell.lassp.houle.RngPack

  Class and Description

  RandomElement

  RandomElement is an abstract class that encapsulates uniform random number
  generators.

  RandomSeedable

  `RandomSeedable` is an abstract class that extends the
  `RandomElement` class to include the ability to automatically
  generate a valid `long` seed from the clock.

- Overview
- Package
- Class
- Use
- Tree
- Deprecated
- Index
- Help

- Prev
- Next

- Frames
- No Frames

- All Classes
